# Cardiovascular Depression in Rats Exposed to Inhaled Particulate Matter and Ozone: Effects of Diet-Induced Metabolic Syndrome

**DOI:** 10.1289/ehp.1307085

**Published:** 2013-10-29

**Authors:** James G. Wagner, Katryn Allen, Hui-yu Yang, Bin Nan, Masako Morishita, Bhramar Mukherjee, J. Timothy Dvonch, Catherine Spino, Gregory D. Fink, Sanjay Rajagopalan, Qinghua Sun, Robert D. Brook, Jack R. Harkema

**Affiliations:** 1 Department of Pathobiology and Diagnostic Investigation, and; 2 Center for Integrative Toxicology, Michigan State University, East Lansing, Michigan, USA; 3 Department of Biostatistics, and; 4 Department of Environmental Health Sciences, School of Public Health, University of Michigan, Ann Arbor, Michigan, USA; 5 Department of Pharmacology and Toxicology, Michigan State University, East Lansing, Michigan, USA; 6 Davis Heart and Lung Research Institute, The Ohio State University College of Medicine, Columbus, Ohio, USA; 7 Department of Internal Medicine, University of Michigan, Ann Arbor, Michigan, USA

## Abstract

Background: High ambient levels of ozone (O_3_) and fine particulate matter (PM_2.5_) are associated with cardiovascular morbidity and mortality, especially in people with preexisting cardiopulmonary diseases. Enhanced susceptibility to the toxicity of air pollutants may include individuals with metabolic syndrome (MetS).

Objective: We tested the hypothesis that cardiovascular responses to O_3_ and PM_2.5_ will be enhanced in rats with diet-induced MetS.

Methods: Male Sprague-Dawley rats were fed a high-fructose diet (HFrD) to induce MetS and then exposed to O_3_, concentrated ambient PM_2.5_, or the combination of O_3_ plus PM_2.5_ for 9 days. Data related to heart rate (HR), HR variability (HRV), and blood pressure (BP) were collected.

Results: Consistent with MetS, HFrD rats were hypertensive and insulin resistant, and had elevated fasting levels of blood glucose and triglycerides. Decreases in HR and BP, which were found in all exposure groups, were greater and more persistent in HFrD rats compared with those fed a normal diet (ND). Coexposure to O_3_ plus PM_2.5_ induced acute drops in HR and BP in all rats, but only ND rats adapted after 2 days. HFrD rats had little exposure-related changes in HRV, whereas ND rats had increased HRV during O_3_ exposure, modest decreases with PM_2.5_, and dramatic decreases during O_3_ plus PM_2.5_ coexposures.

Conclusions: Cardiovascular depression in O_3_- and PM_2.5_-exposed rats was enhanced and prolonged in rats with HFrD-induced MetS. These results in rodents suggest that people with MetS may be prone to similar exaggerated BP and HR responses to inhaled air pollutants.

Citation: Wagner JG, Allen K, Yang HY, Nan B, Morishita M, Mukherjee B, Dvonch JT, Spino C, Fink GD, Rajagopalan S, Sun Q, Brook RD, Harkema JR. 2014. Cardiovascular depression in rats exposed to inhaled particulate matter and ozone: effects of diet-induced metabolic syndrome. Environ Health Perspect 122:27–33; http://dx.doi.org/10.1289/ehp.1307085

## Introduction

Metabolic syndrome (MetS) is a group of risk factors for developing type II diabetes mellitus and cardiovascular disease that includes at least three of the following conditions concurrently: hypertension, central obesity, elevated fasting glucose, high serum triglycerides and low circulating high-density lipoprotein (National Cholesterol Education Program 2001). MetS affects approximately 32% of the U.S. population and is expected to grow to 34% by 2020 (Ford et al. 2010). Among dietary and other lifestyle factors, high intake of fructose has been proposed to contribute to the the development of MetS, although a causal relationship is controversial (Feinman and Fine 2013; Stanhope 2012). Fructose-sweetened beverages can increase visceral adiposity and induce insulin resistance and dyslipidemia after only 10 weeks of consumption (Stanhope et al. 2009). Fructose-induced MetS has been effectively modeled in rats, where hypertension, hypertriglyceridemia, and insulin resistance are induced after an 8- to 10-week period of fructose supplementation (Patel et al. 2009; Tran et al. 2009).

Recent epidemiological studies have indicated that elevated ambient concentrations of fine particulate matter (aerodynamic diameter ≤ 2.5 μm; PM_2.5_) or carbon monoxide are linked to greater decreases in heart rate variability (HRV), an index of autonomic balance, in MetS subjects compared with healthy subjects (Min et al. 2009; Park et al. 2010). In addition, we have recently reported that small increases in urban ambient PM_2.5_ can decrease insulin sensitivity in healthy subjects (Brook et al. 2013), suggesting that PM_2.5_ may contribute to MetS etiology or to the progression from MetS to diabetes. Given the high prevalence of MetS, the cardiovascular and metabolic health risk of exposure to ambient pollutants may be substantial.

Compared with the link between MetS and air pollution exposure, the link between morbidity and mortality due to diabetes and exposure to ambient air pollution has been more documented (Ostro et al. 2006; Zanobetti and Schwartz 2011). For example exposure to PM_2.5_ is associated with enhanced vascular reactivity (O’Neill et al. 2005) and cardiac function abnormalities (Baja et al. 2010) in diabetics. Acute cardiovascular responses to ozone (O_3_) exposure are also exaggerated, with increased heart rate (HR) (Hampel et al. 2012) and decreased blood pressure (BP) (Hoffmann et al. 2012). In addition, obesity and hypertension, common comorbidities of both MetS and diabetes, are themselves susceptibility factors for adverse responses to PM_2.5_ (Dubowsky et al. 2006).

Health complications from air pollutant exposures have been modeled in rodents with hypertension and various other chronic cardiovascular diseases, but comparable studies of experimental metabolic disorders are lacking. In the present study we used a novel model of high-fructose feeding to induce the MetS phenotype and then exposed these metabolically challenged rodents to different air pollutant atmospheres to test the hypothesis that adverse cardiovascular effects of air pollution would be exacerbated by the MetS. Our exposure regimens consisted of O_3_, PM_2.5_, and O_3_ plus PM_2.5_, an approach that is consistent with calls for more research on the health risks of multipollutant atmospheres (Johns et al. 2012). We analyzed changes in HR, HRV and BP to assess the effects of exposure on vascular, cardiac, and autonomic function in the face of diet-induced metabolic dysregulation.

## Materials and Methods

*Animals*. Eight-week-old male Sprague Dawley rats, weighing 250–275 g (Charles River Laboratories, Portage, MI) were fed either a normal diet (ND; 8640 Teklad 22/5 Rodent Diet; Harlan Laboratories, Madison, WI), or a high-fructose diet (HFrD; 60% fructose by mass; TD.89247; Harlan Laboratories) to induce the MetS phenotype. After 8 weeks on ND or HFrD, rats were transported to AirCARE 1, a mobile air research laboratory parked in Dearborn, Michigan, and randomly assigned to one of four exposure groups: filtered air (FA), O_3_, PM_2.5_, or O_3_ + PM_2.5_, for a total of eight experimental groups (*n* = 7–8/group for metabolic outcomes; *n* = 4 for telemetry end points). Rats were housed individually in polycarbonate shoebox-type cages with corncob bedding and had access to food and autoclaved and filtered water. Inhalation exposures were conducted 8 hr/day (0730–1530 hours) for 9 consecutive weekdays (week 1, Monday–Friday; week 2, Monday–Thursday) to capture hourly and daily variation of ambient PM. All rats were sacrificed 24 hr after the last exposure (week 2, Friday). Food and water were removed during exposures. Study protocols were approved by the Institutional Animal Care and Use Committee of Michigan State University (MSU) to ensure human treatment of animals; MSU is accredited by the Association for Assessment and Accreditation of Laboratory Animal Care.

*Exposure to O_3_ and PM_2.5_*_._ We conducted inhalation exposures in AirCARE 1, parked at Salinas Elementary School in Dearborn, Michigan, during the summer of 2011. This urban industrial location is also a stationary air pollution monitoring site operated by the Michigan Department of Environmental Quality and experiences some of the highest annual PM_2.5_ concentrations in Michigan (Michigan Department of Environmental Quality 2012). The site is located within 5 km of iron/steel production facilities, a coke oven, an oil refinery, a sewage sludge waste incinerator, a coal-fired power plant, and major highways.

Concentrated PM_2.5_ was generated from ambient PM_2.5_ using a Harvard-type fine particle concentrator and Hinners whole body animal exposures chambers as previously described in detail (Harkema et al. 2004). O_3_ was generated using an OREC O_3_ generator (Model V1, ultraviolet light method; OREC, Akron, OH), and O_3_ concentration was targeted at 0.5 ppm. Because of exposure chamber configurations, exposures to FA and O_3_ + PM_2.5_ were conducted during different weeks (25 July–4 August 2011) than exposures to O_3_ or PM_2.5_ alone (15–25 August 2011).

*Exposure characterization*. We conducted air quality monitoring on both concentrated and ambient PM_2.5_ samples using methods previously described by Morishita et al. (2006). Briefly, integrated and concentrated PM_2.5_ mass samples were determined during each 8-hr exposure period using Teflon filters. Ambient and concentrated PM_2.5_ mass was also measured continuously (5-min interval). Particle compositions of acidity, sulfate, nitrate, ammonium ion, elemental carbon, and organic carbon were determined. Concentrations trace elements were assessed using high-resolution inductively coupled plasma mass spectrometry (ICP-MS; ELEMENT2; Thermo Finnigan, San Jose, CA). Further details of the exposure assessment are provided in Supplemental Material, p. 2.

*Cardiovascular telemetry*. Ten weeks before exposures, rats were surgically implanted with PhysioTel Multiplus transmitters (C50-PXT; Data Sciences International, St. Paul, MN) that emit radio signals of electrocardiograms (ECG) and BP. Transmitters were placed with ECG leads terminating in a lead-II configuration in order to sample cardiac parameters, and the pressure catheter was placed in the aorta via the femoral artery. Telemetry receivers (RLA3000; Data Sciences International) were modified and affixed inside individual cages in exposure chambers. Because of space limitations, we used four rats from each dietary group for collection of BP and ECG waveforms. We collected 30-sec-long data streams every 5 min during exposures (0730–1530 hours), and during nonexposure times in evenings (0000–0500 hours) and weekends (0730–1530 hours). Automated ECG analysis (Ponemah software; Data Sciences International) allowed for R-wave detection on a beat-to-beat basis. We use R-R intervals for all normal beats to calculate HR and time-domain measures of HRV; standard deviation of the normal-to-normal beats (SDNN), an indicator of overall autonomic tone; and the square root of the mean squared differences of successive normal-to-normal intervals (RMSSD), an estimate of parasympathetic tone.

*Metabolic end points*. We collected serum from fasted animals for determination of glucose, insulin, and triglycerides levels. Briefly, blood glucose was measured using a Bayer Contour glucometer (Bayer, Whippany, NJ). Serum insulin levels were measured using the ultrasensitive rat insulin ELISA kit (Crystal Chem, Downers Grove, IL), and triglycerides were measured using the L-Type Triglyceride M Assay (Wako Diagnostics, Richmond, VA). Insulin resistance was determined using the homeostatic model assessment of insulin restance (HOMA-IR), which assesses the ratio of fasting blood glucose and insulin and is routinely used for human clinic assessment (Brook et al. 2013) as described further in Supplemental Material (pp. 1–2).

*Statistical analyses*. We used a two-way factorial design with repeated measures over time, consisting of two diet groups (HFrD and ND) and four exposure groups (O_3_, PM_2.5_, O_3_ + PM_2.5_, and FA). We implemented repeated measures analyses using a linear mixed model with nested random effects of time within date to estimate the effects of exposure and diet and their interaction on each outcome for the entire time series observed over a 9-day study period. An autoregressive model of order 1 correlation structure was considered in the analysis because the correlation between observations decreases if the observations are further apart in time. To reduce the skewness of the HRV measures, we natural log–transformed the SDNN and RMSSD after adding 1.

We used linear mixed models for the effects of exposure and diet for each of the 9 days in the study period to obtain daily results, in which the random effect is time only. Statistical analysis was performed using both R 2.12.1 (http://www.r-project.org/) and SAS 9.2 (SAS Institute Inc., Cary, NC). Criteria for significance were set at *p* ≤ 0.05 for all parameters.

## Results

*Exposure characterization*. The average daily chamber concentrations of PM_2.5_ were 356 ± 87 μg/m^3^ (mean ± SD) for the group exposed to PM_2.5_ alone, and 441 ± 65 μg/​m^3^ for the O_3_ + PM_2.5_ coexposures. Average O_3_ concentrations were 0.485 ± 0.042 ppm for the group exposed to O_3_ alone, and 0.497 ± 0.030 ppm for the O_3_ + PM_2.5_ coexposures. Data on the major components of PM_2.5_ (sulfates, nitrates, carbon, ammonium, and organic matter), and of trace elements is available in Supplemental Material, Tables S1 and S2, respectively. Relative contributions from major components and trace elements in PM_2.5_ were typical for urban industrial southwestern Michigan in summer months that we have documented in previous field exposures (Harkema et al. 2004; Rohr et al. 2011).

**Figure 1:**
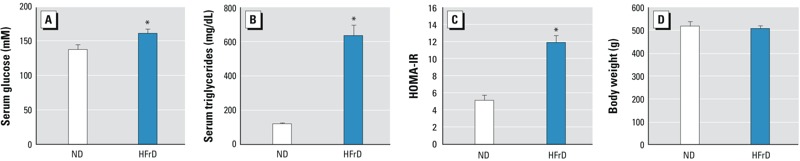
Metabolic responses in rats fed ND versus HFrD for 10 weeks. Serum glucose (*A*), serum triglycerides (*B*), insulin resistance as measured by HOMA-IR (*C*), and body weight (*D*) were determined as described in “Materials and Methods.” Data are expressed as mean ± SE (*n* = 8/group). *p < 0.05 compared with ND, by *Student’s t-test*.

*Metabolic end points*. Ten weeks of the HFrD induced hyperglycemia, dyslipidemia with elevated serum triglycerides, and insulin resistance (Figure 1). Body weights were not different between dietary groups.

*Blood pressure*. Baseline mean arterial pressure (MAP) prior to inhalation exposures was significantly greater in HFrD rats compared with ND rats (116.3 ± 8.9 mmHg vs. 103.4 ± 11.4 mmHg, respectively), which is consistent with previous findings in rats fed the HFrD (Patel et al. 2009). MAP was unaffected by FA exposure (102.5 ± 9.5 mmHg). In ND rats, only coexposure to O_3_ + PM_2.5_ affected MAP, with a modest but significant decrease of 3.2 mmHg that was similar to the response in HFrD rats (Table 1). In comparison, HFrD rats were much more sensitive to exposure-related change in MAP, with reductions in blood pressures of 6.9 and 7.6 mmHg for O_3_ and PM_2.5_, respectively (Table 1). With the exception of a modest increase in diastolic pressure in ozone-exposed ND rats (2.4 mmHg), diet- and exposure-related changes in systolic and diastolic pressures were similar to those observed with MAP (Table 1).

**Table 1:** Change in cardiovascular and autonomic parameters during 9-day exposure to O_3_, PM_2.5_, or O_3_ + PM_2.5_.

Parameter	Diet	Experimental exposure		
		O_3_	PM_2.5_	O_3_ + PM_2.5_
MAP (mmHg)	ND	0.45 ± 0.81	–0.07 ± 0.93	–3.20 ± 0.82*
	HFrD	–6.87 ± 0.89*^,^**	–7.65 ± 0.89*^,^**	–4.9 ± 0.95*
Systolic BP (mmHg)	ND	–2.09 ± 1.09	–1.20 ± 1.09	–2.93 ± 1.10*
	HFrD	–13.82 ± 1.20*^,^**	–8.83 ± 1.20*^,^**	–7.92 ± 1.28*^,^**
Diastolic BP (mmHg)	ND	2.37 ± 0.66*	0.77 ± 0.66	–3.01 ± 0.67*
	HFrD	–1.9 ± 0.72*^,^**	–6.73 ± 0.73*^,^**	–2.75 ± 0.77*
HR (bpm)	ND	–6.19 ± 2.14*	–15.24 ± 2.16*	–10.75 ± 2.17*
	HFrD	–40.94 ± 2.34*^,^**	–34.68 ± 2.34*^,^**	–24.67 ± 2.35*^,^**
SDNN (msec)	ND	3.87 ± 0.74*	–2.51 ± 0.75*	–6.54 ± 0.75*
	HFrD	1.27 ± 0.87**	1.52 ± 0.81**	–1.43 ± 0.81**
RMSSD (msec)	ND	6.23 ± 1.01*	–2.46 ± 1.01*	–8.7 ± 1.01*
	HFrD	2.35 ± 1.1*^,^**	2.82 ± 1.1*^,^**	–1.46 ± 1.1**
Data (mean ± SE) are the estimated changes in BP, HR, and HRV (SDNN and rMSSD) in response to diet and experimental exposures compared with exposure to FA (*n *= 4/per group). Effect estimates were determined using linear mixed modeling as described in “Materials and Methods.” *p < 0.05 compared with the corresponding FA group. **p < 0.05 compared with the corresponding ND group.				

**Figure 2:**
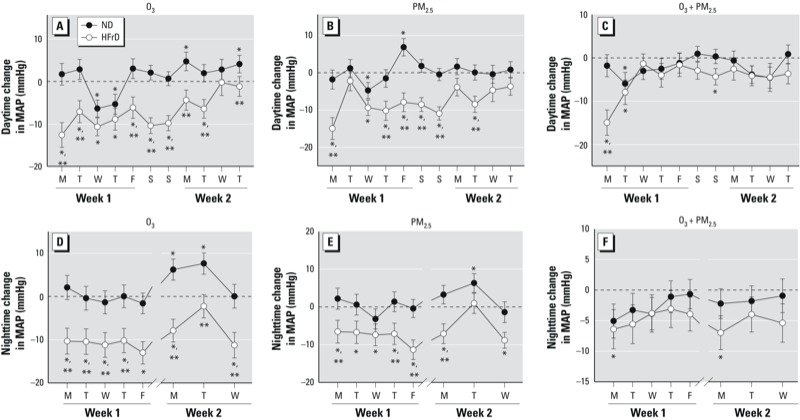
Daily changes in MAP (mmHg) during the daytime (weekdays and during non­exposure hours on weekends; *A*,*B*,*C*) and nighttime (non­exposure hours during evenings; *D*,*E*,*F*) in rats fed ND or HFrD and exposed to O_3_ (*A*,*D*), PM_2.5_ (*B*,*E*), or O_3_ + PM_2.5_ (*C*,*F*). Data are expressed as mean ± SE, analyzed by linear mixed models (*n* = 4/group). Dotted lines represent FA-exposed rats. *p < 0.05 compared with FA-exposed rats on the same diet. **p < 0.05 compared with rats fed ND.

Acute decreases in MAP of 10–15 mmHg were evident in HFrD-rats during the first day of exposure to O_3_ or PM_2.5_ and were sustained during the first week of exposures (Figure 2A,B). Interestingly, depressed MAP was sustained over the weekend when rats were not exposed. By the last day of exposure, the MAP of pollutant-exposed HFrD rats was not different from that measured in rats exposed to FA, suggesting that adaptive responses to repeated exposures had occurred in these animals. Modest increases in MAP in ND rats were sporadically observed during exposure to O_3_ (Figure 2A, second week) and to PM_2.5_ (Figure 2B, first week). During evenings postexposure when animals were resting in polycarbonate boxes, exposure-related changes were sustained in both ND and HFrD rats (Figure 2D,E). In HFrD rats, MAP was dramatically decreased during the first 2 days of coexposure to O_3_ + PM_2.5_ (Figure 2C), but only minor changes occurred during the remaining exposure period. In contrast, the O_3_ + PM_2.5_ coexposure had no effect in ND rats.

*HR*. Baseline HR prior to the start of air pollutant exposures was significantly greater in HFrD rats than in ND rats (329 ± 27 bpm vs. 300 ± 35 bpm, respectively). Elevated HR is a consistent finding in human MetS (Grassi et al. 2009; Guize et al. 2008), although it has not been previously reported in HFrD rats. HR was unaffected during FA exposures (299.9 ± 34.9). Decreases in HR were induced by exposures to O_3_, PM_2.5_, or O_3_ + PM_2.5_ regardless of diet (Table 1). Greater declines, however, occurred in HFrD rats compared with ND rats for all exposures, with reductions in HR of up to 12.5% (~ 40 bpm) during O_3_ exposure.

**Figure 3:**
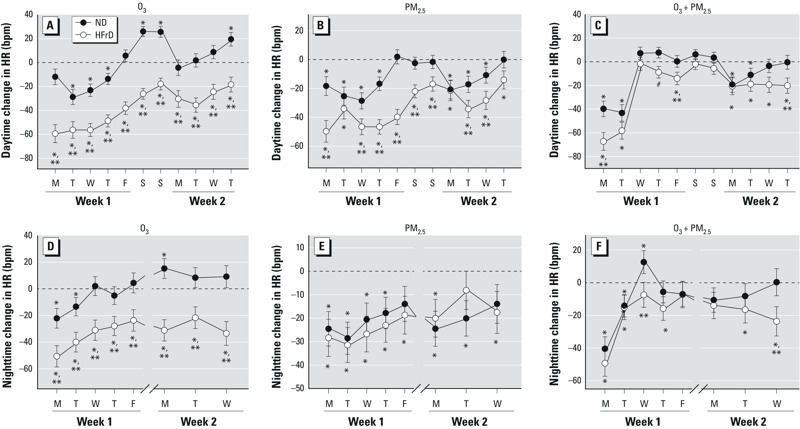
Daily changes in HR (bpm) during the daytime (weekdays and during non­exposure hours on weekends; *A*,*B*,*C*) and nighttime (non­exposure hours during evenings; *D*,*E*,*F*) in rats fed ND or HFrD and exposed to O_3_ (*A*,*D*), PM_2.5_ (*B*,*E*), or O_3_ + PM_2.5_ (*C*,*F*). Data are expressed as mean ± SE, analyzed by linear mixed models (*n* = 4/group). Dotted lines represent FA-exposed rats. *p < 0.05 compared with FA-exposed rats fed the same diet. **p < 0.05 compared with rats fed ND.

We observed acute drops in HR during the first few days of exposure to O_3_ or PM_2.5_ in all rats (Figure 3A,B), but by the end of the first week, HR returned to control levels in ND—but not in HFrD—rats. During the weekend when animals were not being exposed to air pollutants, ND and HFrD had divergent carryover responses from exposures. Decreased HR during O_3_ or PM_2.5_ exposure was sustained in HFrD rats (~ 20 bpm), despite the rats breathing FA during this time. By comparison, HR increased by up to 25 bpm over the weekend in ND rats that had been exposed to O_3_ (Figure 3A). During the second week of exposures, significant depression in HR was again induced by O_3_ or PM_2.5_ exposures in HFrD rats, and by PM_2.5_ exposure to ND rats. In contrast, HR increases in O_3_-exposed ND rats were not significant during the second week except on the final day of exposure (Figure 3A).

Coexposure to O_3_ + PM_2.5_ produced dramatic (~ 73 bpm) decreases in HR in all rats during the first two days of exposure (Figure 3C), but HR rapidly returned to control levels by the third day. No exposure-related effects were detected during the weekend. Depressed HR was again induced during the second week of exposures; however, it remained attenuated compared to exposures to O_3_ or PM_2.5_ alone.

Changes in HR induced by all exposure scenarios were sustained during postexposure evenings (Figure 3D,E,F). Reduced HR was evident after a single 8-hr exposure (i.e., evening of day 1), and reductions with repeated exposures were of similar magnitude as changes measured during exposures.

**Figure 4:**
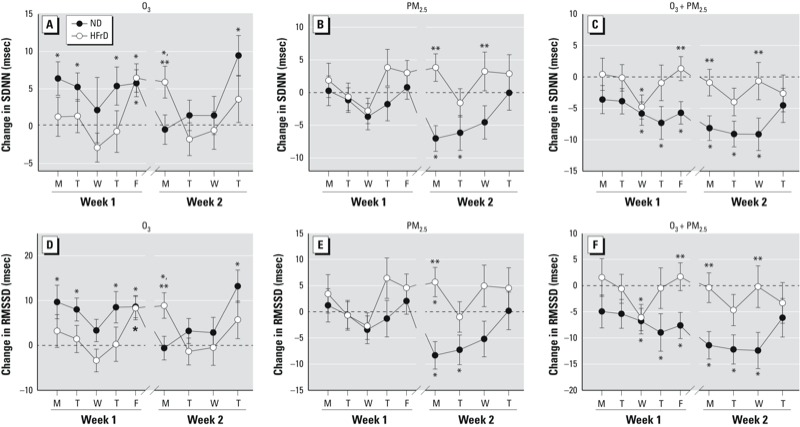
Daily changes in SDNN (*A*,*B*,*C*) and RMSSD (*D*,*E*,*F*) in rats fed ND or HFrD and exposed to O_3_ (*A*,*D*), PM_2.5_ (*B*,*E*), or O_3_ + PM_2.5_ (*C*,*F*). Data are expressed as mean ± SE, analyzed by linear mixed models (*n* = 4/group). Dotted lines represent FA-exposed rats. *p < 0.05 compared with FA-exposed rats on the same diet. **p < 0.05 compared with rats fed ND.

*HRV*. Baseline SDNN and RMSSD prior to exposures were significantly greater in ND rats (14.2 ± 18.5 msec and 12.1 ± 14.4 msec, respectively) than in HFrD rats (10.7 ± 15.1 msec and 8.9 ± 16.9). These results are consistent with the prevalence of low HRV in human MetS (Liao et al. 1998) and in HFrD-induced MetS in rats (Moraes-Silva et al. 2013). In ND-fed rats, O_3_ exposures resulted in increased SDNN and RMSSD (33% and 46%, respectively), whereas PM_2.5_ resulted in decreases (21% and 18%) (Table 1). In comparison exposure to O_3_ + PM_2.5_ resulted in a 60% decrease in HRV. In contrast to ND rats, HRV responses in HFrD rats exposed to O_3_ or PM_2.5_, compared with FA, were less pronounced, with no changes elicited in SDNN and modest increases in RMSSD (Table 1). In ND rats, the effects of O_3_ were predominantly during the first week of exposure, whereas PM_2.5_-elicited effects occurred during the second week (Figure 4). During O_3_ + PM_2.5_ exposures, daily decreases in both SDNN and RMSDD were similar each day. In HFrD rats, O_3_ caused increased HRV on two days, while PM_2.5_ caused elevated RMSSD on a single day of the 9 days of exposure.

## Discussion

Results of this study clearly demonstrate an interaction between diet and pollutant exposure: Rats with HFrD-induced MetS had enhanced depression of HR and BP during inhalation exposure to O_3_ or PM_2.5,_ compared with similarly exposed ND-fed rats. Exposure-induced decreases of BP and HR occurred during the first day of exposure in HFrD rats, persisted with repeated exposures, and remained depressed during nonexposure periods (evenings and weekends). Responses in healthy rats fed ND were less robust and showed adaptation with repeated exposures. In contrast to their enhanced sensitivity for BP and HR responses, HFrD rats had muted HRV responses compared with ND rats. This is the first study to describe perturbations of normal cardiovascular responses to air pollutant exposures in a rodent model of MetS.

Inhalation exposure–induced depression of HR in rodents has been documented for O_3_ (Farraj et al. 2012; Uchiyama and Yokoyama 1989), diesel exhaust (Lamb et al. 2012), and ambient PM_2.5_ (Kamal et al. 2011). Furthermore, acute exposure of laboratory rodents to O_3_ (Uchiyama and Yokoyama 1989) or ambient PM_2.5_ (Cheng et al. 2003) also triggers a drop in BP, suggesting that inhalation of a variety of airborne toxicants can lead to cardiovascular depression in laboratory animals. Activation of sensory irritant receptors has long been proposed to mediate both the pulmonary and cardiovascular responses to a range of substances, including O_3_ and components of PM (Alarie 1973). In the present study, we found that these responses are exaggerated and prolonged in rats with MetS. The enhanced cardiovascular depression in rats exposed to O_3_ is consistent with recent reports of ambient O_3_–associated bradycardia in infants (Peel et al. 2011) and depressed systolic pressure in persons with diabetes (Hoffmann et al. 2012). Both decreased and increased BP have been reported in diabetics in response to ambient PM_2.5_, with these differences due to the time of the response relative to the start of exposure (acute vs. lag responses, respectively; Hoffmann et al. 2012; Schneider et al. 2010).

Unlike rats fed ND, rats with MetS either experienced delayed adaption or failed to adapt to single-pollutant exposures. After the acute responses of bradycardia and respiratory depression induced by O_3_ or PM inhalation, rodents normally become refractory to repeated exposures (Hamade and Tankersley 2009; Watkinson et al. 2001). Not only did O_3_-exposed ND rats adapt by day 4, they also displayed increased—rather than decreased—BP and HR after several days. ND rats showed rapid adaptation of HR during O_3_ + PM_2.5_ coexposure and significant drops in RMSSD, which suggests a diminution of parasympathetic dominance with repeated coexposures. In contrast, HFrD rats, which also displayed rapid adaptation during coexposures, showed less HRV response and had weaker responses in SDNN and RMSSD to all of the pollutant exposures. This pattern of response in HFrD rats is consistent with cardiovascular autonomic neuropathy described in diabetics who display impairment in autonomic control (Pop-Busui 2012).

In a previous study, Farraj et al. (2012) reported that acute depression of HR during inhalation of 0.8 ppm O_3_ in rats was accompanied by ECG profiles with prolonged PR interval and ST depression, alterations that are consistent with delayed atrioventricular conduction. Similar ECG results have been reported with bradycardia during inhalation of diesel exhaust or metal-rich PM (Farraj et al. 2011; Lamb et al. 2012). Although we did not assess electrical conduction in the present study, exposures to O_3_, PM_2.5_, or O_3_ + PM_2.5_ may initiate ECG alterations that were further modified in HFrD rats to decrease heart rate. Cardiac conduction abnormalities, including atrioventricular block, are common in the diabetic heart (Movahed 2007), but cardiac electrical conduction in HFrD models has not been extensively studied. Fructose can slow excitation–contraction coupling and prolong relaxation in cardiomyocytes (Ren et al. 1997), and rats fed a HFrD have been reported to develop cardiac inflammation, myocardium remodeling, and ventricular dilation (Patel et al. 2009). In separate analysis of the epicardial adipose tissue from our rats, we found exposure-related infiltration of macrophages that was associated with expression of inducible nitric oxide synthase, tumor necrosis factor α, and leptin (Sun et al. 2013). Further studies are needed to describe the nature of exposure-related ECG changes in rats with cardiomyopathies.

Susceptibility of HFrD-fed rats to exaggerated autonomic responses to inhaled pollutants may be centrally mediated at cardiovascular regulatory sites in the brain. Stimulation of α_2_ adrenoceptors in the anterior hypothalamus leads to decreased BP and HR that is greater in HFrD rats than in control rats (Mayer et al. 2007). Supersensitivity of hypothalamic adrenoceptors in response to hyperglycemia has been hypothesized to underlie this effect. We have previously shown elevated production of norepinephrine, an α_2_-receptor agonist, in the paraventricular nucleus of the anterior hypothalamus after a single exposure of rats to PM_2.5_ (Sirivelu et al. 2006). Repeated exposures to PM_2.5_ resulted in sustained increases in hypothalamus norepinephrine in insulin-resistant, obese JCR rats, but not in healthy lean control rats (Balasubramanian et al. 2013). This relationship is consistent with our current observation of dramatic drops in HR and BP during the first few days of exposure that are sustained in HFrD but not ND rats. Thus, the enhanced and sustained stimulation of sympathoinhibitory α_2_ adrenoceptors by chronic release of norepinephrine in the hypothalamus could explain the robust and relatively more persistent cardiovascular depression in exposed HFrD rats.

It is notable that exposure to either O_3_ or PM_2.5_ alone resulted in cardiovascular depression of a similar magnitude and time-course, yet, by comparison, the effects of the multipollutant exposure to O_3_ + PM_2.5_ were blunted. We hypothesize that the combination of particulate- and oxidant-induced toxicity may stimulate defensive and adaptive responses more quickly and strongly than those elicited by exposure to a single pollutant. However, because our PM exposures with and without O^3^ were conducted at different times, a direct comparison of these groups to determine the interaction between O_3_ and PM_2.5_ was not possible.

Translation of our results from this HFrD model may be limited to individuals with MetS who have a high intake of dietary fructose. Individuals with MetS associated with high-fat or high-calorie diets or genetic predisposition may have different responses to air pollutant exposure. A second limitation of our study is that we used rodents in inhalation studies to model human responses. Although several research groups also reported cardiovascular depression in animals from O_3_ and PM exposure, many human studies report hypertensive and increased HR responses to ambient pollutant exposure (Hampel et al. 2012). We have mentioned some important exceptions in diabetics and infants above (Hoffmann et al. 2012; Peel et al. 2011; Schneider et al. 2010), where elevations in ambient O_3_ were associated with decreased HR and BP, similar to what we describe in HFrD rats. Finally, our results should be interpreted with caution because of the limited number of animals per group, even though statistical power was sufficient for our analysis.

## Conclusion

This is the first report of dysregulation of normal cardiac, vascular, and autonomic responses to inhalation exposure to O_3_ and PM_2.5_ in rats with HFrD-induced MetS. Exaggerated depression and delayed adaptation of BP and HR to air pollutants in HFrD rats was accompanied by the lack of adjustment in autonomic control as measured by HRV. This suggests that underlying cardiovascular and autonomic neuropathies caused by MetS or metabolic disorders such as diabetes may promote inappropriate cardioregulatory responses to repeated expsoure to ambient air pollutants. Specific alterations in central versus local neurotransmission, cardiac tissue remodeling, and production of soluble mediators in HFrD rats during inhalation exposure remain to be identified. With one-third of the U.S. population compromised by MetS, the health impact of oxidant and particulate air pollutants in this sensitive population is likely to be significant. Future research using this model of HFrD-induced MetS will contribute to the development of prevention and intervention strategies to protect this susceptible population from the adverse cardiovascular effects of multipollutant atmospheres.

## Supplemental Material

(270 KB) PDF
