# Supplementary material for: Cardiovascular Depression in Rats Exposed to Inhaled Particulate Matter and Ozone: Effects of Diet-Induced Metabolic Syndrome
Source: Environ Health Perspect. 2013 Oct 29;122(1):27–33. doi: 10.1289/ehp.1307085 (PMC3888573; doi:10.1289/ehp.1307085)
Supplement: (270 KB) PDF [file ehp.1307085.s001.508.pdf]

Environmental Health Perspectives doi: 10.1289/ehp1307085

Supplemental Material

Cardiovascular Depression in Rats Exposed to Inhaled Particulate Matter and Ozone: Effects of Diet-Induced Metabolic Syndrome

James G. Wagner, Katryn Allen, Hui-yu Yang, Bin Nan, Masako Morishita, Bhramar Mukherjee, J. Timothy Dvonch, Catherine Spino, Gregory D. Fink, Sanjay Rajagopalan, Qinghua Sun, Robert D. Brook, and Jack R. Harkema

Table of Contents:

Metabolic Endpoints

Exposure Assessment

Analytical Methods

Reference

Supplemental Material, Table S1. Major Components in Concentrated $PM_{2.5}$

Supplemental Material, Table S2. Trace Elements in Concentrated $PM_{2.5}$

Metabolic Endpoints

Animals were fasted for 16 hours prior to blood draws for blood glucose and serum insulin. Isoflourane was used to anesthetize the animals and blood was drawn from the ventral artery on the tail using a 27-gauge syringe. Blood glucose was measured using a Bayer Contour®USB glucometer (Tarrytown, NY). Blood was collected for serum separation, and insulin levels were measured using the ultrasensitive rat insulin ELISA kit from Crystal Chem, Inc. (Downers Grove, IL) per the manufacturer's instruction. The homeostatic model assessment of insulin resistance (HOMA-IR) was calculated using the following equation:

HOMA – IR = [glucose (mMol/L) x insulin (μU/mL)]/22.5

Serum for triglyceride analysis was collected at necropsy and analyzed using the Wako Diagnostics (Richmond, VA) L-Type Tg M assay per the manufacture's instruction.

Exposure Assessment

CAPs samples were collected on Teflon and quartz filters (Gelman Sciences, Ann Arbor MI) by attaching Teflon filter packs (URG Corp., Chapel Hill, NC) to the back of the animal exposure chamber for the duration of each 8-h exposure period. Sodium carbonate-coated backup filters were placed downstream of the Teflon filters to correct for nitrate volatilization losses. The volume of air drawn through each particulate sampling train was determined using a calibrated dry test meter (DTM) (Schlumberger, Owenton, KY). TEOMs 1400ab were also used to monitor both ambient $PM_{2.5}$ and CAPs concentrations.

Analytical Methods

Sample handling, processing, and analysis took place in a Class 100 ultra-clean laboratory at the University of Michigan Air Quality Laboratory, designed for ultra-trace element analysis with an emphasis on low-level environmental determinations. Measurements including field blanks, filter-lot blanks, replicate analyses, and externally certified standards were incorporated into all analyses for quality assurance and quality control purposes. Gravimetric analysis was performed using a microbalance (MT-5 Mettler Toledo, Columbus, OH) in a temperature/humidity-controlled clean laboratory as described in Federal Reference Method (USEPA 1997). CAPs samples collected on quartz filters were stored at -40ºC after sampling and analyzed for carbonaceous aerosols by a thermal-optical analyzer using NIOSH Method 5040 (Sunset Labs,

Forest Grove, OR). Teflon filters and carbonate-coated backup filters were extracted in Milli-Q ultrapure water. Extracts were then analyzed for anions and cations by chromatography (Model DX-600, DIONEX, Sunnyvale, CA). Samples collected from Teflon filters were analyzed for a suite of trace elements using high-resolution inductively coupled plasma-mass spectrometry (ICP-MS) (ELEMENT2, Thermo Finnigan, San Jose, CA).

Reference

USEPA, 1997. Reference method for the determination of fine particulate matter as PM2.5 in the atmosphere. EPA 40 CFR Pat 50. Washington, DC.

Supplemental Material, Table S1. Major components in concentrated $PM_{2.5}$.

**$PM_{2.5}$ Component ($\mu g/m^3$)**	**Exposure 1** (**$PM_{2.5}$** *alone*)	**Exposure 2** (**$PM_{2.5}$** + *Ozone*)
Mass	441 $\pm$ 196	356 $\pm$ 261
Organic Matter	114 $\pm$ 57	148 $\pm$ 81
Elemental Carbon	5 $\pm$ 3	10 $\pm$ 5
Sulfate	99 $\pm$ 86	70 $\pm$ 122
Nitrate	29 $\pm$ 20	15 $\pm$ 6
Ammonium	54 $\pm$ 47	20 $\pm$ 19
Urban Dust	38 $\pm$ 12	52 $\pm$ 23

Total $PM_{2.5}$ mass and major components were determined in exposure chambers during exposure to $PM_{2.5}$ or to $PM_{2.5}$ + O_3. Data are expressed as mean ± SD for 9 observations.

Supplemental Material, Table S2. Trace Elements in Concentrated $PM_{2.5}$.

Element (ng/m^3)	Exposure 1	Exposure 2
Na	774 ± 517	1,522 ± 1,433
Mg	1,614 ± 889	2,644 ± 1,462
AL	9,821 ± 408	1,349 ± 613
P	263 ± 67	294 ± 77
S	39,308 ± 33,415	29,842 ± 47,410
Ca	6,663 ± 4,143	10,470 ± 422
Ti	46.6 ± 21.3	76.1 ± 33.4
V	10.4 ± 9.3	10.2 ± 9.7
Cr	51 ± 23	70 ± 52
Mn	228 ± 118	438 ± 347
Fe	3,522 ± 1,238	5,517 ± 2,696
Co	1.01 ± 0.27	1.3 ± 0.64
Ni	15.8 ± 8.2	21.0 ± 11.7
Cu	97 ± 34	142 ± 60
Zn	888 ± 874	2,484 ± 2,710
K	1,490 ± 386	1,895 ± 928
As	11.7 ± 7.1	14.6 ± 13.8
Se	24.1 ± 17.6	33.6 ± 48.2
Rb	3.1 ± 1.9	7.3 ± 7.8
Sr	36.6 ± 23.5	51.1 ± 18.8
Mo	5.0 ± 1.8	8.6 ± 6.5
Cd	2.4 ± 3.2	16.2 ± 28.3
Sb	13.5 ± 6.0	25.5 ± 27.9
Ba	181 ± 91	293 ± 115
La	1.81 ± 1.57	2.55 ± 2.42
Ce	2.36 ± 2.08	2.51 ± 1.11
Pb	73 ± 50	277 ± 381

Content of trace elements in $PM_{2.5}$ were determined in exposure chambers during exposure to $PM_{2.5}$ or to $PM_{2.5}$ + O_3. Data are expressed as mean ± SD for 9 observations.
